# eRFSVM: a hybrid classifier to predict enhancers-integrating random forests with support vector machines

**DOI:** 10.1186/s41065-016-0012-2

**Published:** 2016-06-30

**Authors:** Fang Huang, Jiawei Shen, Qingli Guo, Yongyong Shi

**Affiliations:** 1 Bio-X Institutes, Key Laboratory for the Genetics of Developmental and Neuropsychiatric Disorders (Ministry of Education) and the Collaborative Innovation Center for Brain Science, Shanghai Jiao Tong University, Shanghai, 200030 People’s Republic of China; 2 Shanghai Changning Mental Health Center, Shanghai, 200042 People’s Republic of China; 3 Department of Psychiatry, The First Teaching Hospital of Xinjiang Medical University, Urumqi, 830054 People’s Republic of China; 4 The Bio-X Little White Building, Shanghai Jiao Tong University, No.55 Guang Yuan Xi Road, Shanghai, 200030 China

**Keywords:** Enhancer, Hybrid classifier, Algorithm

## Abstract

**Background:**

Enhancers are tissue specific distal regulation elements, playing vital roles in gene regulation and expression. The prediction and identification of enhancers are important but challenging issues for bioinformatics studies. Existing computational methods, mostly single classifiers, can only predict the transcriptional coactivator EP300 based enhancers and show low generalization performance.

**Results:**

We built a hybrid classifier called eRFSVM in this study, using random forests as a base classifier, and support vector machines as a main classifier. eRFSVM integrated two components as eRFSVM-ENCODE and eRFSVM-FANTOM5 with diverse features and labels. The base classifier trained datasets from a single tissue or cell with random forests. The main classifier made the final decision by support vector machines algorithm, with the predicting results of base classifiers as inputs. For eRFSVM-ENCODE, we trained datasets from cell lines including Gm12878, Hep, H1-hesc and Huvec, using ChIP-Seq datasets as features and EP300 based enhancers as labels. We tested eRFSVM-ENCODE on K562 dataset, and resulted in a predicting precision of 83.69 %, which was much better than existing classifiers. For eRFSVM-FANTOM5, with enhancers identified by RNA in FANTOM5 project as labels, the precision, recall, F-score and accuracy were 86.17 %, 36.06 %, 50.84 % and 93.38 % using eRFSVM, increasing 23.24 % (69.92 %), 97.05 % (18.30 %), 76.90 % (28.74 %), 4.69 % (89.20 %) than the existing algorithm, respectively.

**Conclusions:**

All these results demonstrated that eRFSVM was a better classifier in predicting both EP300 based and FAMTOM5 RNAs based enhancers.

**Electronic supplementary material:**

The online version of this article (doi:10.1186/s41065-016-0012-2) contains supplementary material, which is available to authorized users.

## Background

Transcription regulation in human genes is a complex process. Systematic and precise identification of these regulatory DNA elements, especially enhancers [17] is a prerequisite to understand gene expression in both healthy and diseased cells [9]. More and more studies indicate that mutations in enhancers are associated with human diseases, such as cancers [7, 15], cardiovascular diseases [21] and immunological diseases [27].

Enhancers increase the transcriptional output in cells manifesting distinct properties, which are summarized as follows: (a) enhancers are distal regulatory elements, which may locate 20kb or further away from transcription start sites, (b) they can activate gene transcription by recruiting transcription factors (TFs) and their complexes, (c) they may be enriched with chromatin modifications, such as monomethylation of histone H3 lysine 4 (H3K4me1) and the acetylation of histone H3 lysine 27 (H3K27ac), (d) they can initiate RNA polymerase II transcription, producing a new class of non-coding RNAs called enhancer RNAs (eRNAs) [1], (e) they are tissue specific and merely conservative functioning in different spaces and stages. Thus, single experimental validation of them seems to be a time-consuming and costly task. Predicting enhancers based on conservation analysis of genomic sequences also doesn’t work well [23]. With the development of high-throughput sequencing technologies, the advanced computational tools make this task possible in the big data era.

Machine learning algorithms [31] were used to predict enhancers with chromatin immune precipitation sequencing (ChIP-Seq) datasets [10, 30], such as the chromatin modification loci and the TF binding sites (TFBs) [24, 31]. Single classifiers used supervised learning algorithms, e.g., CSI-ANN [13] introduced an artificial neural network approach; RFECS [25] identified enhancers with RF; ChromaGenSVM [12] applied SVMs with a Genetic Algorithm (GA) to optimize the parameters of SVMs; EnhancerFinder [11] used multi-kernel SVMs to predict enhancers in the eukaryotic genome; DEEP [18] used an algorithm combined SVMs with ANN including the components of DEEP-ENCODE and DEEP-FANTOM5.

Some classifiers mentioned above, such as CSI-ANN, ChromaGenSVM, REFCS and EnhancerFinder used ChIP-Seq datasets as features, and were strongly relied on EP300, which was a transcriptional coactivator and could activate gene transcription by combining with TFs, considering EP300 binding sites as enhancers. However, EP300 binding sites are very possible enhancer sites, but not the 100 % real ones [19]. DEEP used the same datasets in predicting EP300 based enhancers as the classifiers mentioned above. It firstly used FANTOM5 datasets as training enhancers, which were reconstructed from enhancer RNAs (eRNAs) datasets using Cap Analysis of Gene Expression (CAGE) [14]. However, it used DNA sequence features as features not ChIP-Seq datasets in DEEP-FANTOM5. It used SVMs as base classifiers to train datasets from single tissues or cell lines and it used ANN as a main classifier to make the final decision combining the results of the base classifiers. It has been proved that getting the global optimum of ANN is a NP-hard problem. The simple implementing algorithm, back-propagation algorithm, is a heuristic algorithm, which is easy to trap in local optimal solution [26]. Thus, the weakness of DEEP was obvious. In the layer of algorithm, the predicting result was not the global optimum; in the layer of datasets, it didn’t use effective features in predicting eRNAs based enhancers.

In this study, we built a hybrid classifier eRFSVM including eRFSVM-ENCODE and eRFSVM-FANTOM5. We used RF as base classifiers [3, 4], which was a fast and easy- paralleled algorithm good at dealing with unbalanced datasets. For eRFSVM, we could get the global optimum when making the final decision for both RF and SVMs algorithms were P problems [29].

In the process of data pre-processing, to reduce unbalanced ratio, we used a sub-sampling algorithm, and combined it with the k-means method, comprehensively considering the running time for the program and the loss of information in samples. The base classifiers trained datasets from single tissues or cell lines with the RF algorithm, which used 60 % of the datasets for training and the remaining 40 % for testing. With the testing results of base classifiers, we built the main classifier with SVMs. For eRFSVM-ENCODE, we trained datasets of cell lines like Gm12878, Hep, H1-hesc and Huvec, and then tested datasets of Hela and K562 cell lines, with enhancers identified by transcriptional coactivator EP300 binding sets as labels. For eRFSVM-FANTOM5, we trained on datasets of blood, lung, liver, kidney, and then tested on the datasets of adipose, with eRNAs based enhancers as labels.

## Methods

### The ENCODE datasets

The National Human Genome Research Institute (NHGRI) launched a public research consortium named ENCODE, the Encyclopedia of DNA Elements [6], in September 2003, to carry out a project to identify all functional elements in the human genome sequence. The datasets generated by next-generation sequencing technologies or software prediction in the project were available at https://genome.ucsc.edu/ENCODE/, including raw data, alignment data and peak calling data in different kinds of formats. The datasets were used for the studies, such as TF binding sites, histone modifications. In this study, we used the datasets for training and testing in the classifier of eRFSVM-ENCODE from ChIP-Seq experiments of different cell lines. The peak calling datasets of ChIP-Seq experiments from Gm12878, Hep, H1-hesc, Huvec, Hela and K562 in the form of broadpeak were used as features including eleven histone modifications, H2AFZ (Variant of H2A), H3K27ac (Detects of acetylation), H3K27me3 (Detects of trimethylation in Lysine 27), H3K36me3 (Marks of actively transcribed regions), H3K4me1 (Associated with enhancers), H3K4me2 (Marks of promoters and enhancers), H3K4me3 (Associated with active promoters), H3K79me2 (Marks of transcriptional transition regions), H3K9ac (Marks of promoters in chromatin regions), H3K9me3 (Associated with silenced chromatin) and H4K20me1 (Associated with active and accessible regions). Gm12878, Hep, H1-hesc, Huvec, Hela and K562 were cell lines from blood, liver, embryonic stem cell, blood vessel, cervical cancer and blood, respectively. The EP300 datasets were used as labels for the classifiers. The human genome sequences were downloaded from Ensembl (Homo_sapiens.GRCh38.dna).

### The FANTOM5 datasets

The sra or bed format of ChIP-Seq datasets of lung, blood, liver, kidney and adipose including H3K4me1, H3K4me3, H3K9ac, H3K9me3, H3K27ac, H3K27me3 and H3K36me3 from NIH Epigenome Roadmap [2] were used as features for training. The ChIP-Seq of the same tissue were from different kinds of cells and different phases of life. Datasets of kidney were from fetal and adult; datasets of adipose were from mesenchyme and gland; datasets of lung were from left lung and right lung in the fetal; datasets of blood were from K562 and CD4+; datasets of liver were from adult. The FANTOM5 project [1] examines how our genome encodes the fantastic diversity of cell types that make up a human. Using Cap Analysis of Gene Expression (CAGE), studies in FANTOM5 project had mapped the sets of transcripts, transcription factors, promoters and enhancers active in the majority of mammalian primary cell types and a series of cancer cell lines and tissues. Enhancer labels were obtained from FANTOM5 consortium publicly available at http://enhancer.binf.ku.dk/Pre-definedtracks.html.

### Data normalization for histone modifications

Firstly, to process datasets from NIH Epigenome Roadmap, we transformed data in the form of sra [16] to fastq by a software from NCBI. With bwa, we transformed fastq data to sam data. With samtools, we transformed sam data to bed data. Secondly, we used MACS [32] for sharp peak calling and SICER for broad peak calling [22, 28]. ChIP-Seq peaks counted in a 200, 400, 600, 800, 1000-bp window were output.

The resolution of preprocessed histone modification data was 200bp. We used the signal value and normalized it in the formula below to train the datasets.

$$ {x}_i=\frac{x_i-{x}_{\min }}{x_{\max }-{x}_{\min }} $$

### Reduction of imbalanced data ratio

Unbalanced datasets will lead to machine learning bias that classification will tend to put a sample prediction for a majority class sample, which will lead to a lower recognition rate for minority class samples. According to the priori data, enhancers accounts for about 2 % of the genome data as a minority class [19]. So the recognition of minority class samples is the key point of this issue. We used sub-sampling combined with the k-means to get negative samples from the whole genomes and made the ratio between positive and negative datasets as 1:10 [12] to reduce the imbalanced ratio.

### Base classifier algorithm (The random forests algorithm)

RF [3, 4] are ensemble of decision trees, which are based on information gain, the computation formula are presented as:

$$ \inf {o}_A(D)=-{\displaystyle \sum_{i=1}^m{p}_i{ \log}_2\left({p}_i\right)} $$

$$ gain(A)= \inf o(D)- \inf {o}_A(D) $$

The step of RF can be represented as:

- use bootstrap to extract k samples from the original training sets with N samples for k times,
- establish k decision trees,
- vote according to the classification results of all decision trees, the voting results called confidence-score can be described as:

$$ confidence- score=\frac{tree\_ number(positive)}{tree\_ number(total)} $$

RF overcomes the limitation of over-fitting of decision trees, with advantage of fast and simple, tolerance to noise and abnormal value. In this study, we used the signal values of histone modifications in each sample as features and computed confidence-score of all decision trees. The confidence-score was used as a feature for the main classifier.

### Main classifier algorithm (The support vector machines algorithm)

Recently, the support vector machines (SVMs) [8] is an algorithm for classification or regression, which aims to separate the positive data and negative data through a hyperplane with datasets mapped to high dimensions with maximal margin. The algorithm tries to minimize structural risk and is less vulnerable to the over-fitting problem. Given training vectors x_i_ ∈ R^n^, i = 1,…, n, in two classes, and a vector of label y ∈ R^m^ such that y_i_ ∈(−1,1) [20], SVMs solves a quadratic optimization problem by means of a kernel function like polynomial and radial basis function (RBF). The principle of SVMs can be described as follows:

$$ \min \frac{1}{2}\left|\right|w\left|\right|{}^2+C{\displaystyle \sum_{i=1}^n{\varepsilon}_i} $$

$$ s.t.\kern1.6em {y}_i\left({w}^T\varPhi \left({x}_i\right)+b\right)\ge 1-{\varepsilon}_i,{\varepsilon}_i\ge 0 $$

$$ {\xi}_i\ge 0,i=1,\dots, \kern0.1em m $$

For any testing instance x, the decision function (predictor) is described as follows:

$$ f(x)=\operatorname{sgn}\left({w}^T\phi (x)+b\right) $$

Practically, we need only *k*(*x*, *x* ') = *ϕ*(*x*)^*T*^ *ϕ*(*x* '), the kernel function, to train with SVMs. The RBF kernel is used in our experiments:

$$ k\left(x,x\hbox{'}\right)= \exp \left(-\gamma \left|\right|x-x\hbox{'}\left|\right|{}^2\right) $$

With the RBF kernel, there are two parameters to be determined in the SVMs: C and γ. To get the good generalization ability, we conduct a validation process to decide parameters. The procedure is described as the following:

- consider a grid space of (C, γ) with C ∈ {0,2,4,…,50} and γ∈{0,2,4,…,200},
- for each parameter pair (C, γ) in the search space, conduct 10-fold cross validation (CV) on the training sets,
- choose the parameter pair (C, γ) that leads to the lowest CV balanced error rate,
- use the best parameter pair to create a model as the predictor.

### Implementing eRFSVM-ENCODE

Firstly, with the normalized features from Gm12878, Hep, H1-hesc, Huvec, we built base classifiers with RF algorithm separately, using 60 % of each datasets for training and the rest 40 % for testing. RF was not sensitive to input parameters, thus, we just used the default parameters for each classifier. We calculated the precision, F-score of each base classifiers. With the results of base classifiers, we used SVMs to merge the testing results of the four single classifiers. With the confidence-score multiplying the F-score of each classifier as features of main classifier, we used 10-k fold cross-validation. SVMs was sensitive to the parameters, so we used the Gaussian kernel as default and searched for the best C and γ with the grid search algorithm. With base classifiers training on datasets from Gm12878, Hep, H1-hesc, Huvec, we tested them on K562 and hela datasets. The time complexities of RF and SVMs are *O* (N_features_*M_samples_) [8] and *O* (N_features_*M^3^_samples_) respectively, the whole complexity of eRFSVM is *O* (N_features_*M^3^_samples)_, which means the running time is proportional to the number of feature and the third power of sample. The computational time for base classifier training on specific cell line was 40 min and training on the four datasets merged with SVMs for 8 h and testing on K562 for 2 h in a server with 4 CPU cores and 48 GB RAM (Intel Xeon 2.4 GHz). The program can be downloaded in http://analysis.bio-x.cn/SHEsisMain.htm.

### Implementing eRFSVM-FANTOM5

**Fig. 1:**
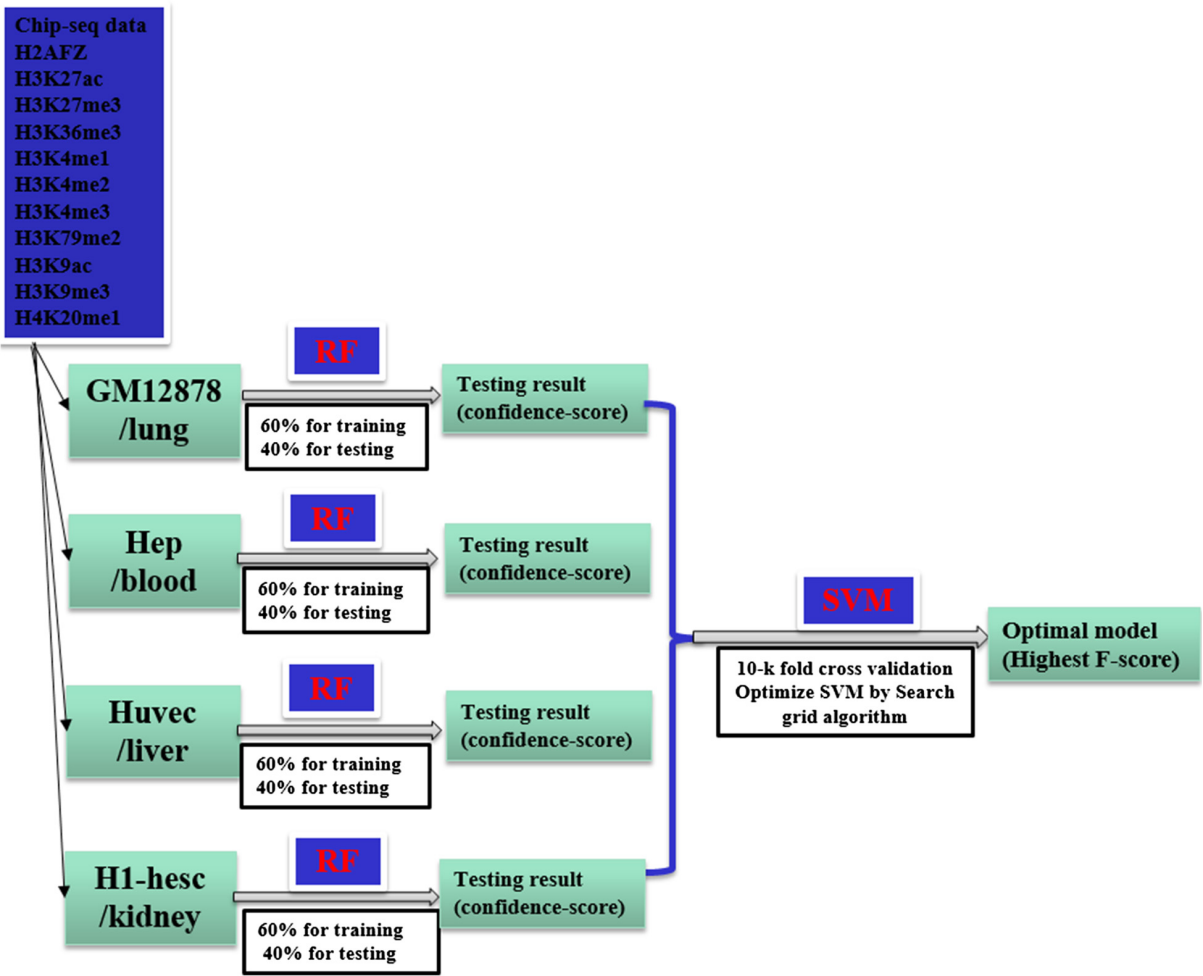
The overview of eRFSVM (Different RF classifiers are made as base classifiers and SVMs classifier is made as main classifier)

We trained the datasets from blood, lung, kidney, liver and tested them on the adipose as the same framework (Fig. 1) that eRFSVM-ENCODE used. The computational time for model training on a specific cell line was 10 min and training on the four datasets merged with SVMs for 1 h and testing on adipose for 5 min.

### Performance evaluation of classifiers

The trained classifiers return confidence scores between 0 and 1 for a combined histone modification profiles. These scores are then transformed to a binary state indicating ‘enhancer’ or ‘not enhancer’ by choosing a cut-off. For each combination of profiles, the existence of regulatory element is considered positive (P) or negative (N) otherwise. True (T) means that the predicted functional states are enhancers, and false (F) implies otherwise. The notations of TP, FP, TN and FN combined these labels to return the number of each class. The performance evaluation of classifiers is made according to the following formulas:

$$ \Pr ecison=\frac{TP}{TP+FP} $$

$$ \mathrm{R}\mathrm{e} call=\frac{TP}{TP+FN} $$

$$ Specificity=\frac{TN}{TN+FP} $$

$$ Sensitivity=\frac{TP}{TP+FN} $$

$$ F- score=\frac{2*\left( \Pr ecision*\mathrm{R}\mathrm{e} call\right)}{ \Pr ecision+\mathrm{R}\mathrm{e} call} $$

$$ accuracy=\frac{TP+TN}{TP+TP+FP+FN} $$

The predicted confidence scores are transformed into binary predictions by using different cut-offs yielding sensitivity and specificity over the entire score range. ROC plots can well evaluate the performance of classifiers, which display the FP (1-specificity) values on the x-axis, and the TP (sensitivity) values on the y-axis. ROC plots show the direct relationship between the FP and TP rates. The total AUC (area under the curve) for the ROC plot is used to measure the prediction performance of this method.

## Results and Discussion

### Performance of eRFSVM-ENCODE

With the histone modification datasets and EP300 datasets of cultured cell lines in broadpeak format downloaded from ENCODE, we discretized the positive datasets with 200bp as a unit and used sub-sampling [5] and k-means algorithms to get the negative datasets (Additional file 1: Table S1).

For the training steps, the best performed base classifier was hesc, with precision, recall and F-score of 84.53 %, 83.03 % and 83.78 %, respectively. For eRFSVM-ENCODE, we found that the precision, recall and F-score were 92.16 %, 90.70 % and 91.43 %, respectively, which meant that the hybrid classifier fitted better than the base classifiers (Additional file 1: Table S2).

**Fig. 2:**
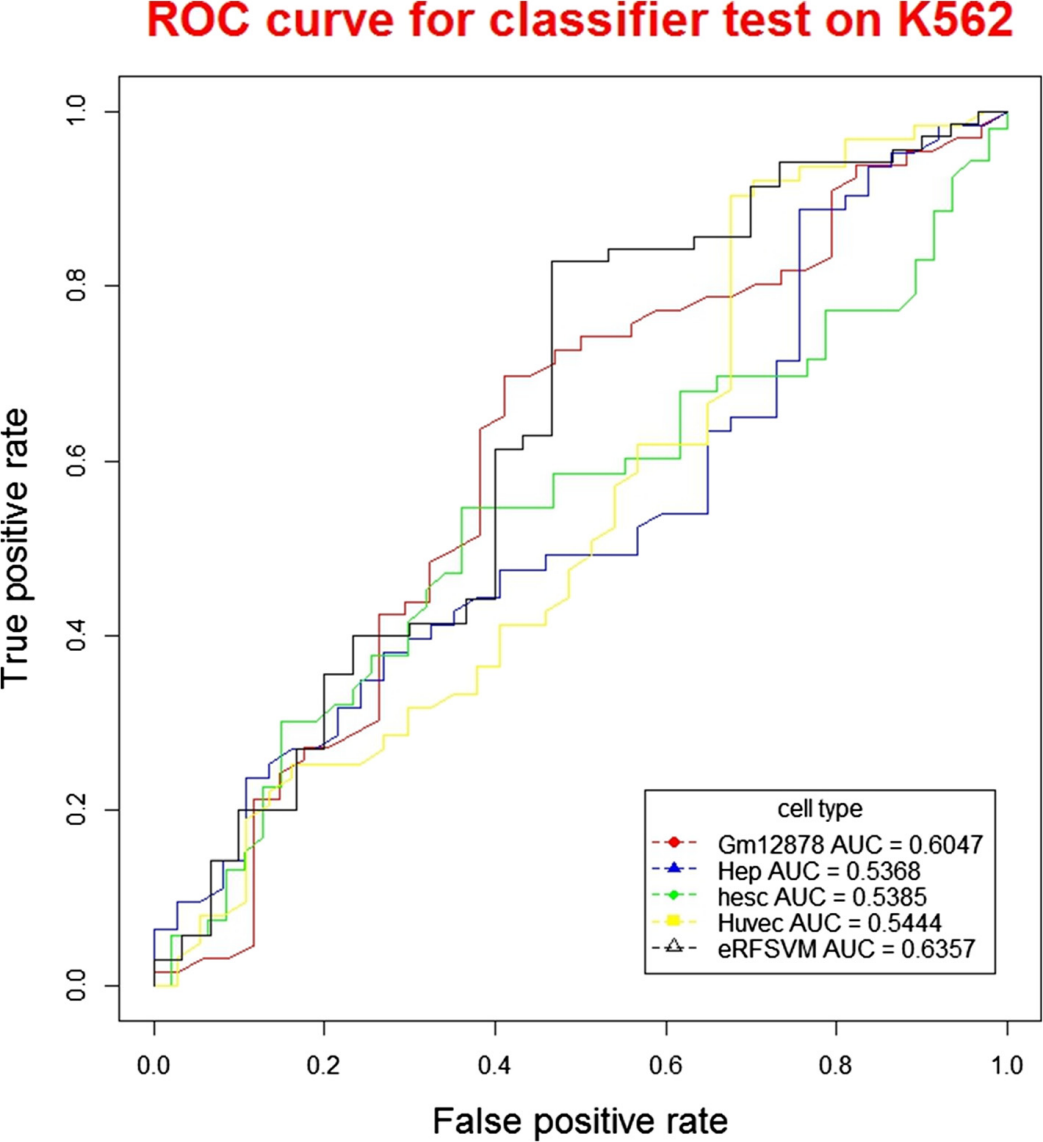
ROC curve for classifier test on K562 (Cross-validation ROC plot of the optimum classifier to predict enhancers in K562 cells)

**Fig. 3:**
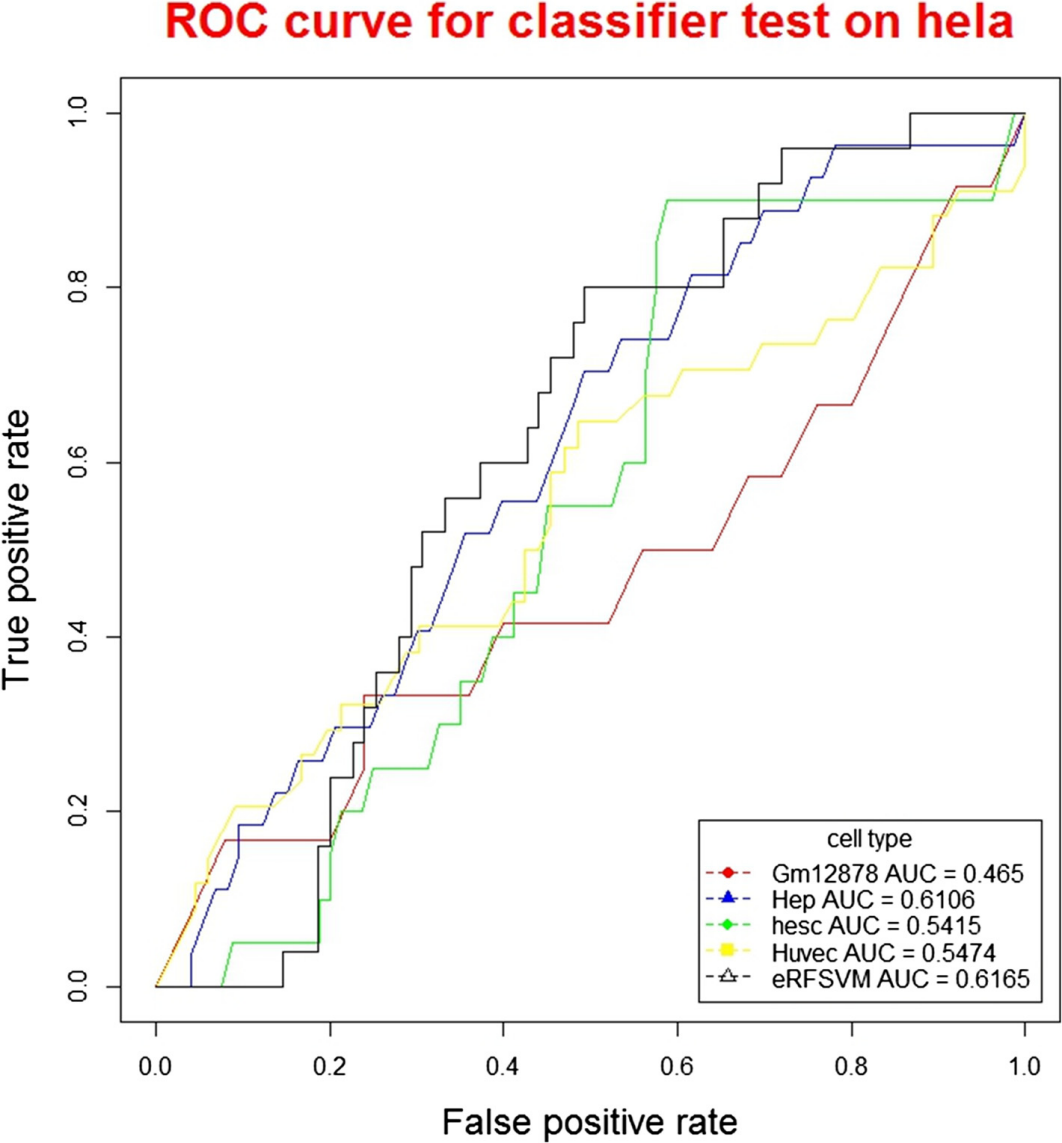
ROC curve for classifier test on hela (Cross-validation ROC plot of the optimum classifier to predict enhancers in hela cells)

When using the classifiers to test on K562 datasets (Table 1), among the base classifiers, GM12878 classifier showed the highest precision (84.39 %); huvec classifier showed the highest recall (6.34 %), F-score (11.76 %) and accuracy (69.79 %). When using classifiers to test on hela datasets, among the base classifiers, hep classifier showed the highest precision (30.24 %) and F-score (6.05 %); GM12878 showed the highest recall (5.47 %) and accuracy (99.33 %). For the hybrid classifier eRFSVM, when testing on K562 datasets, the precision, recall, F-score and accuracy were 83.69 %, 4.92 %, 9.29 % and 69.50 %, respectively, with precision higher than hep and huvec, recall higher than GM12878 and hesc, F-score higher than GM12878 and hesc, accuracy higher than GM12878 and hesc. When testing on hela datasets, the precision, recall and F-score of eRFSVM-ENCODE were 15.35 %, 0.38 % and 0.75 %, with precision higher than GM12878, hesc and huvec, recall higher than hep and huvec, F-score higher than hep and huvec. The ROC curves could comprehensively evaluate the performance of classifiers (Figs. 2 and 3), with higher the AUC value, the better performance of the classifier. The AUC values of GM12878, hep, hesc, huvec and eRFSVM-ENCODE are 0.6049, 0.5369, 0.5385, 0.5444 and 0.6357 for K562 datasets, respectively; 0.4650, 0.6106, 0.5415, 0.5474 and 0.6165 for hela datasets, respectively. eRFSVM-ENCODE showed the highest AUC value in both K562 and hela datasets, which meant the hybrid classifier was of better performance.

**Table 1:** Classifiers testing on K562 and hela

Classifiers	Precision		Recall		F-score		Accuracy	
	K562	hela	K562	hela	K562	hela	K562	hela
Gm12878	84.39 %	13.22 %	0.88 %	3.92 %	1.75 %	6.05 %	68.47 %	99.18 %
hep	83.00 %	30.24 %	5.04 %	0.31 %	9.50 %	0.62 %	69.52 %	99.33 %
hesc	84.00 %	4.73 %	3.66 %	5.47 %	7.01 %	5.07 %	69.19 %	98.63 %
huvec	81.25 %	7.44 %	6.34 %	0.35 %	11.76 %	0.66 %	69.79 %	99.30 %
eRFSVM	83.69 %	15.35 %	4.92 %	0.38 %	9.29 %	0.75 %	69.50 %	99.28 %

### Performance of eRFSVM-FANTOM5

The datasets from NIH Epigenome Roadmap were of different formats. With softwares (see Materials and Methods), we made the peak datasets of different tissues (Additional file 1: Table S3), positive and negative datasets for training of these classifiers (Additional file 1: Table S4).

For the training steps, the best performed base classifier was liver. The mean values of precision, recall and F-score of these base classifiers were 75.42 %, 53.90 % and 61.79 %, respectively. For eRFSVM, we found that the precision, recall and F-score were 90.59 %, 48.16 % and 62.22 %, respectively (Additional file 1: Table S5). The F-score of eRFSVM was higher than that of blood, lung and kidney, which meant eRFSVM fitted better than these base classifiers.

When using the classifiers to test on adipose datasets (Table 2), among the base classifiers, lung classifier showed the highest precision (83.72 %); kidney classifier showed the highest recall (47.31 %), F-score (57.41 %) and accuracy (93.55 %). For the hybrid classifier eRFSVM, when testing on adipose datasets, the precision, recall, F-score and accuracy were 86.17 %, 36.06 %, 50.84 % and 93.38 %, respectively, with precision higher than all the base classifiers, recall higher than blood, liver and lung, F-score higher than blood, liver and lung, accuracy higher than blood, liver and lung.

**Table 2:** Results testing on adipose with ChIP-Seq datasets

Classifiers	Precision	Recall	F-score	Accuracy
blood	55.80 %	31.56 %	40.32 %	91.51 %
liver	80.73 %	27.50 %	41.03 %	92.81 %
lung	83.72 %	11.25 %	19.83 %	91.73 %
kidney	71.83 %	47.81 %	57.41 %	93.55 %
eRFSVM-FANTOM5	86.17 %	36.06 %	50.84 %	93.38 %
SVMs-ANN	65.30 %	28.07 %	39.26 %	92.58 %

**Fig. 4:**
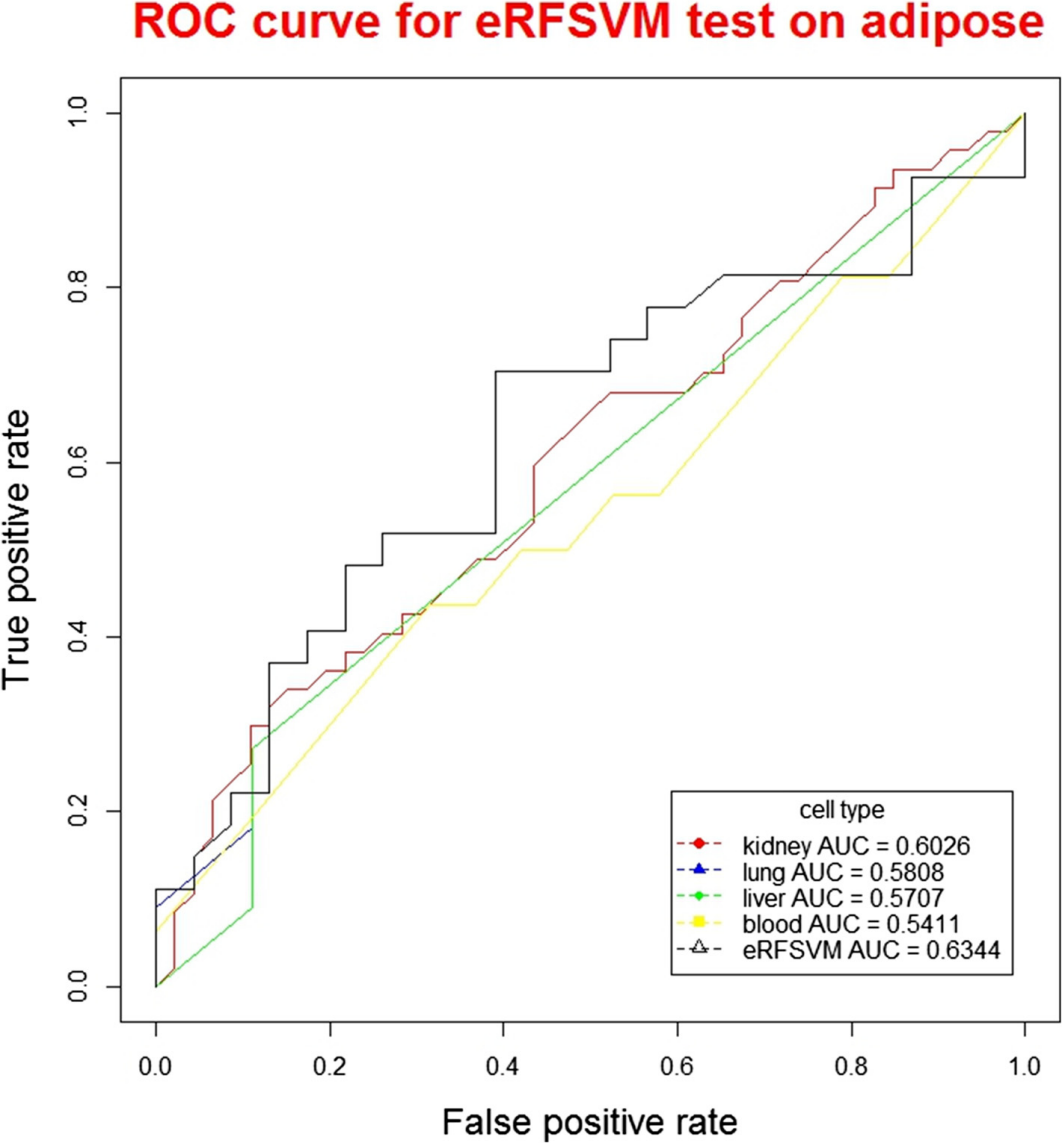
ROC curve for eRFSVM test on adipose (Cross-validation ROC plot of the optimum classifier to predict enhancers on adipose)

The AUC value of blood, liver, lung, kidney and eRFSVM-FANTOM5 are 0.6026, 0.5808, 0.5707, 0.5411 and 0.6344 for adipose datasets, respectively. eRFSVM-FANTOM5 showed the highest AUC value with better performance than base classifiers (Fig. 4).

### Performance camparision of eRFSVM-ENCODE with existing methods

To access the capability of eRFSVM-ENCODE to predict effectively enhancers, we used K562 cell line for testing in a genome wide manner. We compared it with other classifiers, such as CSI-ANN, RFECS and DEEP-ENCODE (Table 3). For K562 cell line, RFECS predicted 130,723,329bp (4.22 %) enhancers; CSI-ANN predicted 34,635,309bp (1.12 %) enhancers; DEEP-ENCODE predicted 28,238,758bp (0.91 %) enhancers; eRFSVM-ENCOE predicted 120,670,200bp (3.89 %) enhancers. eRFSVM-ENCODE and DEEP-ENCODE were hybrid classifiers, having the highest precisions comparing with other methods. eRVSVM-ENCODE had the highest precision of 83.69 % and DEEP-ENCODE had the second highest precision of 83.56 %. The recall and F-score of eRFSVM-ENCODE were both higher than DEEP-ENCODE.

**Table 3:** Comparative performance analysis of the enhancer predictions in K562

Classifiers	Precision	Recall	F-score	Number of predicted enhancer bases (Portion in whole genome)
RFSVM-ENCOE	83.69 %	4.92 %	9.29 %	120,670,200(3.89 %)
CSI_ANN	67.36 %	9.05 %	15.96 %	34,635,309(1.12 %)
RFECS	69.56 %	10.16 %	17.74 %	130,723,329(4.22 %)
DEEP-ENCODE	83.56 %	3.45 %	6.62 %	28,238,758(0.91 %)

### Performance comparison with DEEP-FANTOM5

DEEP-FANTOM5 used SVMs combined ANN (SVMs-ANN) algorithms with DNA sequence features. The precision, recall, F-score and accuracy were 86.17 %, 36.06 %, 50.84 % and 93.38 % using eRFSVM, increasing 23.24 % (69.92 %), 97.05 % (18.30 %), 76.90 % (28.74 %), 4.69 % (89.20 %) using DEEP-FANTOM5, respectively. To compare the features and algorithms in the function of classifiers with eRFSVM-FANTOM5 separately, we built two classifiers in the following.

To compare the effects of different features for classifiers, we used the same algorithm (RF-SVMs) for the classifiers (Tables 2 and 4). DNA sequence features, such as the frequencies of di-nucleotide, tri-nucleotide, tetra-nucleotide, single base were used to make the feature matrix (Additional file 1: Table S6). For the base classifiers, the mean values of precision, recall, F-score were 73.02 %, 29.53 % and 39.65 %, using ChIP-Seq features, respectively; increasing 65.84 % (44.03 %), 8.01 % (27.34 %), 30.0 % (30.50 %) comparing with classifiers using DNA sequence features, respectively (Additional file 1: Table S7). For the hybrid classifiers, the precision, F-score and accuracy were 86.17 %, 50.84 % and 93.38 % using ChIP-Seq features, increasing 2.7 fold (23.61 %), 57.35 % (32.31 %), 6.68 % (87.53 %) comparing with using DNA sequence features, respectively.

**Table 4:** Results tesing on adipose with DNA sequence features

Classifiers	Precision	Recall	F-score	Accuracy
blood	20.77 %	33.75 %	25.71 %	82.27 %
liver	61.84 %	14.69 %	23.74 %	91.42 %
lung	40.07 %	34.06 %	36.82 %	89.38 %
kidney	53.42 %	26.87 %	35.76 %	91.22 %
RF-SVMs	23.61 %	51.25 %	32.31 %	87.53 %
DEEP-FANTOM5	69.92 %	18.30 %	28.74 %	89.20 %

To compare the effects of different algorithms for classifiers, we used the same features (ChIP-Seq datasets) for the classifiers (Tables 2 and 4). The precision, recall, F-score and accuracy were 86.17 %, 36.06 %, 50.84 % and 93.38 % using RF-SVMs, increasing 31.96 % (65.30 %), 7.09 % (28.07 %), 29.59 % (39.26 %), 0.86 % (92.58 %) using SVMs-ANN, respectively.

## Discussion

Previous studies [29] demonstrated that hybrid algorithms could well improve the performance of classifiers, which could be well interpreted in the layer of statistics. A learning algorithm could be considered to search the best performed parameters in the hypothesis space (H), which was near to the real whole space (f). However, the training datasets were not sufficient to the whole real space. Thus, the optimum found in the hypothesis space was very likely not the best in the whole space. The algorithms were easy to trap in local optimum. By combining these hypothesis using hybrid algorithms, classifiers could average these results, leading to much more close to the optimum in the whole real space. In our study, with AUC values to evaluate the performance of classifiers, when testing on the K562, hela and adipsoe datasets, the values of hybrid classifier were 0.6357, 0.6165 and 0.6344, higher than all the base classifiers, verifying that hybrid classifiers were performing better than single classifiers.

The previous methods used EP300 datasets as criteria of enhancers. For eRFSVM-ENCODE, the performance in predicting K562 was better than that in hela. Hela was generated from cervical cancers, much more different from Gm12878 (blood), Hep (liver), H1-hesc (embryonic stem cell) and Huvec (blood vessel), but K562 was from blood, of the same tissue of Gm12878. With more similar cell lines, it would have the better predicting performance. Thus, combining cell lines from much more kinds of tissues would strengthen the predicting performance. We found that F-score of these classifiers was nearly 80 %. However, when testing on other cells, the average F-score of these algorithms was just about 10 %, indicating that the generalization performance of the classifiers were poor. EP300 and enhancers were associated but the relevance was not the same in different tissues, which was one of the reasons leading to the previous classifiers with low generalization performance.

Interpreting enhancers in a variety of angles with biological meaning was an important clue and basis for predicting enhancers with compute methods [19]. Therefore, from a biological perspective, looking for labels with stronger relevance with enhancers was important to build the effective classifiers. FANTOM5 consortium identified enhancers in different tissues and organs in transcription levels. DEEP-FANTOM5 used FANTOM5 enhancers as labels to predict new enhancers. However, DEEP-FANTON5 only used gene sequence features, such as CpG islands, G + C content to create feature matrixes. The average precision of the classifiers was only about 30 % (Additional file 1: Table S8). In this study, we used the ChIP-Seq datasets of different tissues and organs from NIH Epigenome Roadmap as feature matrixes, the average precision rate was about 70 % (Additional file 1: Table S5), which was 2.1 times that of DEEP-FANTOM5. When using eRFSVM-FANTOM5 to predict the adipose datasets, the F-score was 50.84 %, which was close to the F-scores training in blood, kidney and lung. These classifiers performed better compared with classifiers using EP300 datasets as labels.

There are some shortcomings of eRFSVM. Firstly, in the FANTOM5 datasets, only part of the tissues and organs had ChIP-Seq datasets in Roadmap database, therefore, eRFSVM-FANTOM can only predict enhancers in part of tissues and organs. Classifiers suitable for more tissues and cell lines with better generalization performance still need to be developed. Secondly, our knowledge of eRNAs was limited and not all enhancers generate eRNA, thus, enhancers not generating eRNAs may be missed in eRFSVM-FANTOM5. Thirdly, a web based user-friendly tool should be developed in the future.

## Conclusion

In this study, we made a new hybrid classifier eRFSVM using RF combined with SVMs (RF-SVMs) to predict new enhancers in other cell lines or tissues. eRFSVM is more robust than single classifiers with RF with the value of AUC higher than the single classifiers. By training on ENCODE datasets, eRFSVM-ENCOE made 120,670,200bp enhancer predictions covering 3.89 % of the genome. Comparing with state-of-art programs training on ENCODE datasets, eRFSVM-ENCODE made the highest precision of 83.69 % testing on K562 datasets.

For eRFSVM-FANTOM5, with enhancers identified by RNA in FANTOM5 project as labels, we trained datasets on blood, lung, liver and kidney. The best training result of classifiers was liver with a precision of 82.73 %, increasing 2.7 fold compared with DEEP-FANTOM5.

Algorithm RF-SVMs performed better than SVMs-ANN when we used the same features (ChIP-Seq datasets). The precision, recall, F-score and accuracy were 86.17 %, 36.06 %, 50.84 % and 93.38 % using RF-SVMs, increasing 31.96 % (65.30 %), 7.09 % (28.07 %), 29.59 % (39.26 %), 0.86 % (92.58 %) using SVMs-ANN, respectively

ChIP-Seq datasets were better features of classifiers in predicting FANTOM5 enhancers. When using the the same algorithm (RF-SVMs), the precision, F-score and accuracy were 86.17 %, 50.84 % and 93.38 % using ChIP-Seq features, increasing 2.7 fold (23.61 %), 57.35 % (32.31 %), 6.68 % (87.53 %) comparing with using DNA sequence features, respectively.

In conclusion, we provided a better classifier eRFSVM with higher generalization performance both in ENCODE datasets and FANTOM5 datasets comparing with DEEP and other state-of-art classifiers. However, in the FANTOM5 datasets, only part of the tissues and organs had ChIP-Seq datasets in Roadmap, therefore, eRFSVM-FANTOM can only predict enhancers in part of tissues and organs. Classifiers suitable for more tissues and cell lines with better generalization performance still need to be developed.

## Additional file

Additional file 1: Table S1. The datasets for training of cell lines from ENCODE. **Table S2.** Results training on four cell lines. **Table S3.** The peaks of different tissues from Roadmaps. **Table S4.** The datasets for training of different tissues from Roadmap. **Table S5.** Results training on different tissues with ChIP-Seq datasets. **Table S6.** Results training on tissues with DEEP-FANTOM5. **Table S7.** Results training on tissues with sequence features. **Table S8.** Results training on tissues with DEEP-FANTOM5. (DOCX 20 kb)

## Abbreviations

TFs: Transcription factors
eRNAs: Enhancer RNAs
SVMs: Support vector machines
RF: Random forests
ANN: Artificial neural network
CV: Cross validation
CAGE: Cap analysis of gene expression
ROC: Receiver operating characteristic
AUC: Area under roc curve
TP: True positive
FP: False positive
TN: True negative
FN: False negative
TPR: True positive rate
FPR: False positive rate
