# Supplementary material for: eRFSVM: a hybrid classifier to predict enhancers-integrating random forests with support vector machines
Source: Hereditas. 2016 Jun 30;153:6. doi: 10.1186/s41065-016-0012-2 (PMC5226099; doi:10.1186/s41065-016-0012-2)
Supplement: Additional file 1: Table S1. — The datasets for training of cell lines from ENCODE. Table S2. Results training on four cell lines. Table S3. The peaks of different tissues from Roadmaps. Table S4. The datasets for training of different tissues from Roadmap. Table S5. Results training on different tissues with ChIP-Seq datasets. Table S6. Results training on tissues with DEEP-FANTOM5. Table S7. Results training on tissues with sequence features. Table S8. Results training on tissues with DEEP-FANTOM5. (DOCX 20 kb) [file 41065_2016_12_MOESM1_ESM.docx]

eRFSVM: a hybrid classifier to predict enhancers-

integrating random forests with support vector machines

**Tables**

Additional file 1: Table S1. *The datasets for training of cell lines from ENCODE*

| Cell-line | Number of positive bins | Number of negative bins |
| --- | --- | --- |
| Gm12878 | 19362 | 193620 |
| hesc | 8098 | 80980 |
| Hep | 13977 | 139770 |
| Huvec | 32830 | 328300 |

Additional file 1: Table S2*. Results training on four cell lines*

| Classifier | Precision | Recall | F-score | accuracy |
| --- | --- | --- | --- | --- |
| Gm12878 | 79.84% | 81.41% | 80.62% | 96.45% |
| hep | 82.90% | 83.81% | 83.35% | 96.94% |
| hesc | 84.53% | 83.03% | 83.78% | 97.12% |
| huvec | 79.01% | 81.14% | 80.06% | 96.30% |
| eRFSVM | 92.16% | 90.70% | 91.43% | 98.45% |

Additional file 1: Table S3 *.The peaks of different tissues from Roadmaps*

| Histone | blood | adipose | kidney | liver | lung |
| --- | --- | --- | --- | --- | --- |
| H3K4me1 | 125713 | 203609 | 65244 | 61847 | 122035 |
| H3K4me3 | 52422 | 86356 | 45808 | 25722 | 42203 |
| H3K9ac | 51821 | 65245 | 22195 | 20964 | 39265 |
| H3K9me3 | 43231 | 8999 | 4059 | 43699 | 44661 |
| H3K27ac | 58937 | 540477 | 40610 | 44278 | 63421 |
| H3K27me3 | 88069 | 20655 | 7054 | 44278 | 43310 |
| H3K36me3 | 54277 | 265968 | 15914 | 37346 | 48753 |

Additional file 1: Table S4*.The datasets for training of different tissues from Roadmap*

| Classifier | Number of positive bins | Number of negative bins |
| --- | --- | --- |
| Blood | 4067 | 40670 |
| Adipose | 320 | 3200 |
| Lung | 1214 | 12140 |
| Kidney | 545 | 5450 |
| liver | 242 | 2420 |

Additional file 1: Table S5. *Results training on different tissues with ChIP-Seq datasets*

| Classifier | Precision | Recall | F-score | Accuracy |
| --- | --- | --- | --- | --- |
| blood | 68.64% | 37.61% | 48.59% | 92.79% |
| liver | 82.73% | 85.19% | 83.94% | 97.24% |
| lung | 72.45% | 47.46% | 57.35% | 93.41% |
| kidney | 77.84% | 45.35% | 57.31% | 93.74% |
| eRFSVM | 90.59% | 48.16% | 62.22% | 94.47% |

Additional file 1: Table S6. *Results training on tissues with DEEP-FANTOM5*

| Category | Number of features | Description |
| --- | --- | --- |
| Di-nucleotide-frequency | 16 | XY where X,Y ε{A,C,G,T} |
| Tri-nucleotide-frequency | 64 | XYZ where X,Y,Z ε{A,C,G,T} |
| Tetra-nucleotide frequency | 256 | XYZK where X,Y,Z,K ε{A,C,G,T} |
| Single Base frequencies | 4 | X where X ε {A,C,G,T} |
| Aggregate frequencies | 2 | A+T,C+G |

Additional file 1: Table S7 *Results training on tissues with sequence features*

| Classifier | Precision | Recall | F-score | Accuracy |
| --- | --- | --- | --- | --- |
| blood | 48.23% | 5.52% | 9.90% | 90.78% |
| liver | 77.78% | 17.61% | 28.72% | 91.30% |
| lung | 61.04% | 19.05% | 29.04% | 91.40% |
| kidney | 59.40% | 23.65% | 33.83% | 91.41% |
| eRFSVM | 85.68% | 63.53% | 72.96% | 95.60% |

Additional file 1: Table S8 *Results training on tissues with DEEP-FANTOM5*

| Classifier | Precision | Recall | F-score |
| --- | --- | --- | --- |
| brain | 39.41% | 79.72% | 52.75% |
| heart | 33.59% | 76.26% | 46.64% |
| liver | 22.23% | 66.90% | 33.35% |
